# Risk factors and epidemiological characteristics of new neonatal porcine diarrhoea syndrome in four Danish herds

**DOI:** 10.1186/1746-6148-10-151

**Published:** 2014-07-10

**Authors:** Hanne Kongsted, Nils Toft, Jens Peter Nielsen

**Affiliations:** 1 Danish Pig Research Centre, Danish Agriculture and Food Council, Vinkelvej 13, 8620 Kjellerup, Denmark; 2 Department of Large Animal Sciences, HERD – Centre for Herd-oriented Education, Research and Development, University of Copenhagen, Groennegaardsvej 2,1870 Frederiksberg C, Denmark; 3 National Veterinary Institute, Technical University of Denmark, Bülowsvej 27, 1870 Frederiksberg C, Denmark

## Abstract

**Background:**

The epidemiology of New Neonatal Porcine Diarrhoea Syndrome (NNPDS) was studied in four selected herds. A total of 941 new born piglets in 86 litters were evaluated for five consecutive days. NNPDS is a newly emerged syndrome, characterized by diarrhoea within the first week of life, which is un-responsive to antibiotics and not associated with known pathogens. The aetiology behind the syndrome is unknown, and specific risk factors predisposing piglets to develop NNPDS also remain to be determined.

The study evaluated sow and piglet-level risk factors for developing NNPDS and described the epidemiologic characteristics within four herds previously diagnosed with the syndrome. NNPDS was defined as diarrhoea at any time-point during the second to fifth day of life.

**Results:**

NNPDS was observed in a total of 60% (range: 39%-89%) of first parity piglets and 36% (range: 19-65%) of piglets born by mature sows. In total of 26% of piglets had liquid faeces on the day of birth. Approximately half of these piglets developed NNPDS. In the majority of cases (50-70% of cases within herds) symptoms started on the second or third day of life. Piglets in Herd 1 had12.8 times higher probability of developing NNPDS than piglets in Herd 4. First parity piglets had a 4.1 higher probability of developing NNPDS than piglets born by mature sows. Birth weight and faecal consistency on the day of birth were minor risk factors, each significant within one herd.

**Conclusions:**

The most important factors associated with NNPDS were herd of origin and sow-parity. The reason for one of the herds experiencing a considerably more severe outbreak than the others was not explained by factors addressed in this study.

The epidemiological pattern of diarrhoea varied a lot between herds; however, in all herds first parity piglets seemed predisposed. This association may be explained by an infectious background of the syndrome, but further studies are needed to explain this association.

## Background

Neonatal diarrhoea is a well-known disease complex in modern swine production influenced by individual, maternal and environmental factors. The aetiology in specific herd-cases may differ and is often incompletely diagnosed. Until recently, this complexity was not of major practical concern, since most problems could be controlled by vaccination or antibiotics. Around 2008, however, a new syndrome that did not respond to antibiotics or commercial vaccines seemed to emerge ( [1] + Personal communications, S.E. Jorsal, National Veterinary Institute, Technical University of Denmark and B. Svensmark, Pig Research Centre, Danish Agriculture & Food Council, Denmark). The number of affected herds is unknown, but 80% of Danish swine practitioners report to experience these problems [1].

Currently, New neonatal porcine diarrhoea syndrome (NNPDS) refers to a clinical picture with piglets developing diarrhoea that is un-responsive to antibiotics within the first days of life. The suggested piglet-level case-definition is; Non-haemorrhagic diarrhoea during the first week of life without detection of known infectious pathogens, which is characterized by milk-filled stomachs and flaccid intestines at necropsy [2]. This definition is based upon diagnostic examination of 101 Case and Control piglets from the four herds of the current study. Infectious agents which were evaluated and considered not to be involved in the syndrome included; Enterotoxigenic *E. coli*, *Clostridium perfringens* type A and C, *Clostridium difficile*, rotavirus A, coronavirus, *Cystoisospora suis*, *Strongyloides ransomi*, *Giardia spp* and *Cryptosporidium spp*.

Specific factors predisposing piglets to develop NNPDS remain to be discovered. Health problems in sows have previously been associated with diarrhoea in suckling pigs [3-5], but veterinary practitioners do not seem to associate the mastitis-metritis-agalactiae syndrome (MMA) or other sow health conditions with NNPDS [1]. Practitioners report on an association with first parity sows, [1], but this experience needs scientific evaluation.

Insufficient prenatal nutrition or inadequate colostrum supplies are well-known risk factors for neonatal diarrhoea, thus clinical signs suggesting such problems need evaluation in outbreaks of NNPDS. A previous study showed that liquid faeces on the day of birth did not have any negative effects on piglets in these herds in terms of weight gain. Therefore, it was hypothesized that liquid faeces at birth might be a normal phenomenon, unrelated to the syndrome [6]. One of the aims of the present study was to elaborate on this hypothesis, by evaluating if liquid faecal consistency on the day of birth was associated with diarrhoea on the subsequent days.

The primary aim of the study was to investigate sow- and piglet-level risk factors associated with NNPDS. Furthermore, the epidemiological pattern of diarrhoea in terms of prevalence, timing, duration and tendency to cluster within litters was described within the separate herds of the study. Since day one was hypothesized not to be part of the syndrome and piglets were only evaluated until the fifth day of life, NNPDS was defined as diarrhoea at any point during the second to fifth day of life.

## Results

### Description of herds

The four study-herds were conventional indoor production herds and the piglets under study were all Landrace-Yorkshire-Duroc cross-breeds. All herds had experienced problems with neonatal diarrhoea for at least one year. None of the herd-owners were able to point out changes in management connected with the outbreaks. All herds had weekly farrowings and practiced all- in/all-out in farrowing units with appropriate cleaning between farrowing batches. Farrowing crates had partially slatted floors with supplemental heat and cover provided for the piglets. Herd details are given in Table 1.

**Table 1:** Characteristics of the four study-herds

**Herd data**	**Herd 1**	**Herd 2**	**Herd 3**	**Herd 4**
Study period	January 2011	March 2011	May 2011	July 2011
Duration of problems	2 years	>1 year	Since establishment (2 years)	2 years
Herd size (n sows)	900	1250	700	950
Sows per farrowing room	27	40	42	44
SPF^1^-status	Not declared	Not declared	SPF + AP12	SPF
Piglets weaned/sow/year^2^	30.7	27.1	25.4	32.3
1st parity litters (%)^2^	20	22	21	23
Recruitment of gilts	Purchase	Own production	Purchase	Own production
Semen	Purchase	Own boars	Own boars	Purchase
Sow feed^3^	Liquid (residue-free)/Home made	Liquid (residue-free)/Home made	Liquid (residue-free)/Home made	Liquid/Factory made
Routine treatment of piglets^4^	None	None	Amoxicillin at birth	Amoxicillin at castration
Routine treatment of sows^5^	None	None	Oxytocin after farrowing	Oxytocin after farrowing

^1^Specific Pathogen Free – In Denmark most herds participate in a surveillance programme, and are registered for freedom/presence of certain infectious diseases, including PRRS, *M. hyopneumoniae, A. pleuropneumoniae, P. multocida* tox + and *B. hyodysenteriae*. SPF + Ap12 means that the herd is declared free from all SPF diseases except *A. pleuropneumoniae* type 12. ^2^Average calculated from herd-registrations made in a 3 month period prior to investigation. ^3^Feed type used in farrowing period. ^4^Standard antibiotic treatments used in the herds during the first week of life. These treatments were not used during the study. ^5^Any standard medication used on the day of parturition. These treatments were also used during the study.

### Data structure and important herd differences

Altogether, 989 piglets within 86 litters were included at birth. A total of 48 piglets were removed from the data because they were euthanized for necropsy with no history of NNPDS (n = 27), died (n = 20) prior to day five with no history of NNPDS or were hermaphroditic (n = 1). Thus, a total of 941 piglets (227, 245, 216 and 253 from Herds 1, 2, 3 and 4) were included in the analyses. Within Herds 1–4, 5, 10, 5 and 9 first parity sows and 17, 11, 16 and 13 mature sows (2nd-7th parity) were included.

In Herds 1–3, 10% of sows were treated with antibiotics and NSAIDs on day one, whereas this counted for 41% (78% of first parity sows and 15% of mature sows) in Herd 4. All sows in Herd 3 and Herd 4 were treated with oxytocin postpartum. In Herd 2 and Herd 4 a milk formula was given to piglets by drench for supportive care (approximately 10 piglets per herd). Despite the general rule of no antibiotic treatment prior to day 3, in Herd 4, a total of 13 piglets were treated with streptocillin on the second day of life due to arthritis. These piglets were kept in the study, since the treatment was considered of minor importance in the context.

### Clinical findings in piglets on the first day of life

Hollow flanks, rough hair coats, perineal staining and liquid consistency of faeces were relatively prevalent findings in all herds. Liquid consistency of faeces was seen in a total of 26% of piglets (30% of first parity piglets and 23% of piglets born by mature sows). Protruding ribs, fore-knee abrasions and dehydration were low prevalent findings in all herds. A correlation (*r* = 0.67) between faecal consistency and perineal staining was seen. Therefore only faecal consistency was evaluated in the risk-factor analyses. Table 2 presents day one clinical findings in piglets. First parity piglets in Herd 2 were lighter than first parity piglets in the other herds. Furthermore, piglets in this herd had the highest prevalence of hollow flanks, rough hair coats, perineal staining and liquid consistency of faeces.

**Table 2:** Day one clinical findings in 941 piglets from the four herds

**Herd**	**1**	**2**	**3**	**4**	**Total**
**Piglets (first parity/mature sows)**	54/173	117/128	53/163	104/149	328/613
**Birth weight (kg) Mean (sd)**					
**First parity piglets**^**1**^	1.36 (0.3)^a^	1.26 (0.2)^b^	1.34 (0.3)^ab^	1.33 (0.2)^a^	1.31 (0.2)
**Piglets born by mature sows**^**1**^	1.44 (0.3)^a^	1.42 (0.3)^a^	1.47 (0.3)^a^	1.42 (0.3)^a^	1.44 (0.3)
**Clinical appearance (parities combined)**					
**Hollow flanks**	28%	52%	30%	48%	40%
**Rough hair coat**	16%	53%	48%	39%	39%
**Perineal staining**^**2**^	33%	55%	29%	22%	33%
**Liquid faeces**^**2**^	25%	40%	18%	19%	26%
**Protruding ribs**	3%	3%	0.5%	1%	2%
**Fore-knee abrasions**	2%	0.4%	0.5%	5%	2%
**Dehydration**	0%	0%	0%	0%	0%
**Clinical signs of failure to thrive**^3^					
**First parity piglets**	46%	89%	88%	77%	77%
**Piglets born by mature sows**	51%	78%	58%	74%	64%

^1^Different letters within rows indicate significant (P > 0.05) in Welch *t*-test.

^2^Since these variables were correlated, only faecal consistency was evaluated in the risk-factor analysis.

^3^One or more of the following clinical signs; hollow flanks, rough hair coat, perineal staining, liquid faeces, protruding ribs, fore-knee abrasions and dehydration.

### Clinical findings in sows on the day of parturition

Results of sow examinations are summarized in Table 3. Mature sows in Herds 2 and 3 had smaller litter sizes than mature sows in Herd 1 and 4 (mean size approximately 18 piglets vs. 20 piglets). Litter sizes of first parity sows where alike across herds (mean size approximately 15 piglets). The majority of the sows (63/86) did not have any obvious health problems. Certain differences were seen between herds; Herd 1 having more sows suffering from fever and leg problems, Herd 2 having more sows with vulva discharge and Herd 3 having the highest prevalence of clinical mastitis. No obvious link between clinical registrations (made by the first-author of the manuscript) and treatment (carried out by staff-persons) was seen. First parity sows in Herd 4 were very often treated compared to the remaining sows in the study. The staff-persons indication to treat was mastitis.

**Table 3:** Litter sizes, clinical registrations and medical treatment on the day of parturition in 86 sows within the four herds

**Herd**	**1**	**2**	**3**	**4**	**Total**
**n (first parity/Mature)**	5/17	10/11	5/16	9/13	29/57
**Litter size Mean (sd)**	18.6 (2.6)	16.2 (2.8)	17.3 (2.4)	18.3 (3.6)	17.6 (3)
**Litter size 1st parity**^**1**^	15.6 (1.1)^a^	14.4 (2)^a^	16.2 (2.4)^a^	15.8 (2)^a^	15.3 (2)^2^
**Litter size mature sows**^**1**^	19.5 (2.2)^a^	17.9 (2.3)^ab^	17.6 (2.3)^b^	20.1 (3.4)^a^	18.8 (2.7)^2^
**Stillborn Mean (sd)**	2 (1.6)	1 (1.14)	1.7 (1.6)	1.3 (1.3)	1.5 (1.5)
**Fever (≥39.5°C)**	6 (27%)	3 (14%)	2 (10%)	2 (9%)	13 (15%)
**Leg problems**	5 (23%)	1 (5%)	0 (0%)	2 (9%)	8 (9%)
**Mastitis**	0 (0%)	0 (0%)	4 (19%)	1 (5%)	5 (6%)
**Vulva discharge**	0 (0%)	2 (10%)	0 (0%)	0 (0%)	2 (2%)
**Medication**^**2,3**^	2 (9%)	2 (10%)	3 (14%)	9 (41%)	16 (19%)

^1^Different letters within rows indicate significant (P > 0.05) in Welch *t*-test.

^2^Medication included antibiotics and NSAIDs (in Herd 3, two of the sows were treated with NSAIDs only).

^3^Within Herds 1–3, both first parity sows and mature sows were treated. In Herd 4, seven first parity sows (78%) and two mature sows (15%) were treated.

### Occurrence of NNPDS (diarrhoea at day 2–5 of life)

**Figure 1:**
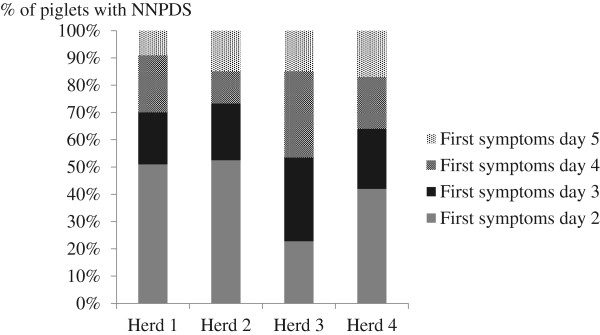
First day with symptoms in 160, 102, 88 and 69 NNPDS affected piglets in Herds 1, 2, 3 and 4.

**Figure 2:**
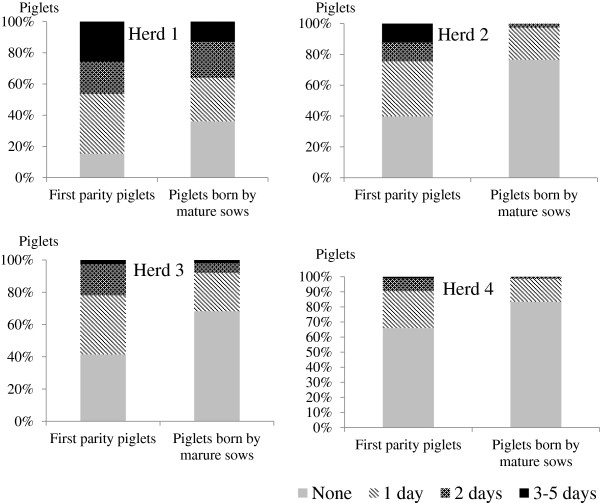
**Duration of NNPDS.** Legend: The figure shows the number of days that piglets were diarrhoeic between day two and five of life. Piglets that were euthanized or died prior to day five of life were not included in these data.

**Figure 3:**
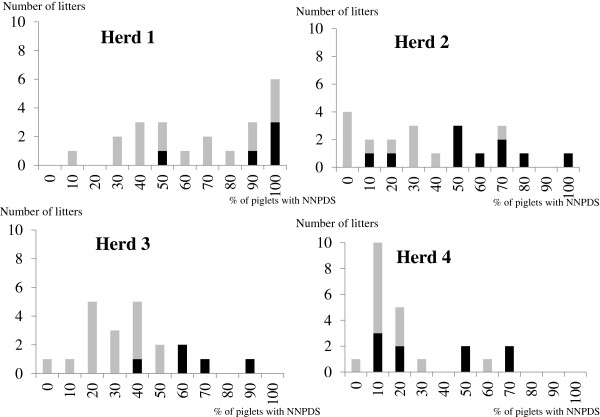
**Prevalence of NNPDS within litters in the four herds.** Legend: First parity litters are presented in black. A 10% prevalence equals approximately one piglet.

In total, 198 (60%) first parity piglets and 221 (36%) of piglets born by mature sows were diarrhoeic at some point between day 2 and five, thus were classified as suffering from NNPDS. The within-herd prevalence of NNPDS and associations between liquid faeces on day one and NNPDS are presented in Table 4. Out of 241 piglets having liquid faeces on the day of birth, a total of 130 (54%) developed NNPDS.In the majority of cases (50-70% of cases within herds), symptoms started on the second or third day of life (Figure 1). The duration of NNPDS (the number of diarrhoeic days between day two and five) in piglets within the four herds is presented in Figure 2. Being affected for one or two days seemed to be the norm, but a few first parity piglets and piglets within Herd 1 experienced symptoms for a longer period. The within-litter prevalence of NNPDS is presented in Figure 3. Both first parity litters and litters of mature sows were affected, but first parity litters were constantly affected and generally had a larger number of diarrhoeic on piglets. In affected first parity litters in average 60% (range: 34-88% in separate herds) of piglets suffered from NNPDS. This counted for an average of 38% (range: 17-62% within separate herds) of piglets in mature parity litters.

**Table 4:** Prevalence of liquid faecal consistency day one and NNPDS in first parity piglets and piglets born by mature sows in the four herds

**Herd**	**1**	**2**	**3**	**4**	**Total**
**First parity piglets (n)**	54	117	53	104	328
Liquid faeces day one	12 (22%)	52 (44%)	10 (19%)	24 (23%)	98 (30%)
NNPDS^1^	48 (89%)	74 (63%)	35 (66%)	41 (39%)	198 (60%)
NNPDS/Liquid faeces day one^2^	10/12	39/52	5/10	12/24	66/98
**Piglets born by mature sows (n)**	173	128	163	149	613
Liquid faeces day one	45 (26%)	47 (37%)	28 (17%)	23 (15%)	143 (23%)
NNPDS^1^	112 (65%)	28 (22%)	53 (33%)	28 (19%)	221 (36%)
NNPDS/Liquid faeces day one^2^	34/45	12/47	12/28	6/23	64/143

^1^NNPDS was defined as diarrhoea at any point during the second to fifth day of life.

^2^Proportion of piglets having liquid faecal consistency day one that developed NNPDS.

Apart from the tendency to affect first parity litters most, no obvious pattern was seen across herds. In Herd 1, piglets with NNPDS seemed to be clustered in litters, whereas in Herd 3 and 4, piglets with NNPDS seemed to be more evenly distributed among litters. In Herd 2, a strong tendency for NNPDS to cluster in first parity litters was observed. Herd 4 stood out as the least affected herd – half of the litters in this herd were either unaffected or had a single piglet with NNPDS only.

### Risk factors for NNPDS at sow level

In the separate parity models (step 1 of the statistical analysis), none of the sow-effects came out significant. Thus, neither litter size, stillborn piglets nor clinical disease in sows were significant risk factors for NNPDS.

The final overall model (step 2 of the statistical analysis) is presented in Table 5. Herd of origin was the most important factor associated with the development of NNPDS, with an OR_PA_ of 12.8 in piglets from Herd 1 compared to piglets from Herd 4. Parity was also an important risk factor with an OR_PA_ of 4.1 in first parity piglets compared to mature parity piglets.

**Table 5:** Results of the overall generalized linear mixed model on NNPDS

**Risk factor**	**Coefficient**	**SE**	**OR**_**PA**_^**1**^	**P-value**
**Intercept**	−1.63			
**Herd^2^**				<0.001
**Herd 4**	0^a^			
**Herd 2**	0.49^a,b^	0.36	1.4	
**Herd 3**	1.11^b^	0.37	2.7	
**Herd 1**	2.68^c^	0.38	12.8	
**Parity**				< 0.001
**2nd-7th**	0			
**1st**	1.54	0.28	4.1	
**Birth weight**				0.003^3^
**Per 100 g**	−0.09	0.03	0.8	
**Day one faecal consistency**				0.006^3^
**Normal**	0			
**Liquid**	0.54	0.19	1.5	
**ICC**^**4**^**litter**	21%			

^1^The population average OR accounts for an average piglet in any litter. ^2^Herds with different letters as superscript are significantly different (α < 0.05) when compared pairwise. ^3^Note: This model did not include interaction effects – therefore these effects are finally evaluated in the herd specific models. ^4^Intra Class Correlation Coefficient (the percentage of total variation in data that is explained by the random litter effect).

Risk factors evaluated included: Herd, parity of sow, number of stillborn, clinical disease in sow, gender, birth weight, faecal consistency, appearance of flanks and appearance of hair coat.

### Risk factors for NNPDS at piglet level

Herd-specific models including the variables that were significant in the overall model were constructed (step 3 in the statistical analysis). Results are presented in Table 6. Parity was the only significant risk factor for NNPDS within all herds (OR_PA_ between 2.4 and 7.9). Random litter effects differed a lot between herds (ICC between 1% (Herd 3) and 39% (Herd1)). Birth weight and faecal consistency day one had similar effects in all herds; however, the effects were only statistically significant within Herd 4 and Herd 2, respectively. The odds of developing NNPDS were increased by 1.1 per 100 g decrease in birth weight (significant in Herd 4). Liquid consistency of faeces on the day of birth increased the odds of developing NNPDS 1.7 times (significant in Herd 2). Since treatment of sows was highly prevalent in Herd 4, a treatment variable (Yes/No) was also tested within this herd. Treatment of sows was not associated with the development of NNPDS (data not shown).

**Table 6:** Results of herd-specific generalized linear mixed models on NNPDS

**Risk factor**	**Herd 1**				**Herd 2**				**Herd 3**				**Herd 4**			
	**Coef.**	**SE**	**OR**_**PA**_^**1**^	***P***	**Coef.**	**SE**	**OR**_**PA**_	***P***	**Coef.**	**SE**	**OR**_**PA**_	***P***	**Coef.**	**SE**	**OR**_**PA**_	***P***
**Intercept**	0.9				−1.9				−0.7				−1			
**Parity**				**0.03**				**<0.01**				**<0.01**				**0.04**
**2nd-7th**	0				0				0				0			
**1st**	2.1	0.99	**6.3**		2.2	0.6	**7.9**		1.4	0.3	**4**		1	0.5	**2.4**	
**Birth weight**				0.11				0.38				0.55				**0.02**
**Per 100 g increase**	−0.1	0.06			−0.1	0.07			−0.1	0.05			−0.2	0.06	**0.8**	
**Faecal consistency**				0.14				**0.04**				0.62				0.12
**Normal**	0				0				0				0			
**Liquid**	0.7	0.45			0.7	0.3	**1.7**		0.2	0.38			0.6	0.4		
**ICC**^**2**^**litter**	39%				26%				1%				15%			

^1^The population average OR accounts for an average piglet in any litter.

^2^Intraclass Correlation Coefficient (the percentage of total variation in data that is explained by the random litter effect).

Variables inserted in the full models included parity, birth weight and fecal consistency day one. OR’s are only displayed for variables with significant effect. Estimates for non-significant variables were extracted from the un-reduced models. Significant associations are presented in bold.

## Discussion

The inclusion of herds for this study was based upon a high prevalence of unexplained neonatal diarrhoea prior to investigation, and intensive diagnostic investigations had suggested that they were all suffering from the emerging syndrome, NNPDS [2]. However, the low prevalence of diarrhoea during the study-period in Herd 4, suggested that this herd was in fact in remission at the point of the study (this was confirmed by follow-up interviews). Since many sows in this herd were medicated on the day of farrowing, the low prevalence of diarrhoea could be hypothetically linked with this. However, data did not support this theory and according to the herd-manager the rate of medication was not higher than in preceding periods with a high prevalence of diarrhoea.

According to interviews with herd-managers, the prevalence of diarrhoea during the study-periods in Herds 2 and 3 was slightly lower than normal, but otherwise reflected their normal situations quite well. A lower prevalence in the study-period can be explained by elements in the study-design made in order to evaluate risk-factors and avoid excess mortality. Thus, restricting litter sizes to a minimum, prohibiting cross-fostering as well as excluding underweight piglets could explain a lower prevalence of diarrhoea than normal. The severe symptoms in Herd 1 matched recordings carried out before and after the study-period. Investigated sow and piglet level risk factors did not give any obvious explanation for this herd to stand out. As previously published, pathological and microbiological findings in piglets from this herd did not differ markedly from findings in the remaining herds [2]. Furthermore, seasonal variations were unlikely to play a role, since Herd 1 was investigated during winter, which is the low season for neonatal diarrhoeas [3,7].

The study intended to describe the epidemiological pattern of NNPDS in terms of prevalence, timing, duration and tendency to cluster within litters. However, important limitations of the study that are relevant in the interpretation of its results need to be mentioned. An obvious limitation of the study was the fact that piglets were only examined for five days. Thus, the definition on NNPDS used in the study was made on practical grounds and should not be interpreted as if NNPDS does not occur beyond the fifth day of life. In fact, 25-50% of piglets within herds started having symptoms on the fourth or fifth day of life, and some of them were probably diarrhoeic beyond the period of examination.

In Herds 1 and 2, the risk of developing diarrhoea was 6–7 fold increased when born by a first parity sow. In the other herds, the association with parity was weaker, but still significant. An association with young sows is in line with previous studies on suckling piglet and neonatal diarrhoea [3,7,8]. Different factors, such as lower levels of colostral antibodies [9], differences in milk composition [10] and stressful behaviour in first parity sows [11] may explain this association. Although no specific microorganism has been identified in the pathogenesis of NNPDS the overrepresentation of first parity litters may be due to lack of specific colostral immunity of a yet unknown infectious agent. It seems intriguing to interpret a tendency to cluster within litters (as seen in Herd 1 and 2) as an indication of the syndrome being of infectious nature. However, inborn (genetic or developmental) and environmental factors are also likely to cluster within litters, thus could also be a part of the explanation.

Obvious health problems in sows were rare and were not statistically associated with development of NNPDS. The lack of association between sow disease and NNPDS is interesting, since it might differentiate this syndrome from previously known neonatal diarrhoeas [3-5]. However, sows were only examined on the day of parturition and may have developed clinical symptoms later that were not taken into account. This study-design was chosen in order to be certain on cause-effect relationships (with piglets developing symptoms on different time-points, it seemed too difficult to evaluate the effect of clinical disease in sows during the whole study-period). In this study, the actual disease effects were probably integrated in the– highly variable – random effects of litters. Thus, the random litter effects probably represented a combination of undiagnosed disease, genetics and local environmental conditions as well as perhaps an infectious agent spreading within litters.

The fact that clinical signs of failure to thrive in piglets at day one were very infrequent (a total of 2% of piglets had protruding ribs) suggested that prenatal nutrition was generally adequate. Furthermore, since hollow flanks on the first day of life were not associated with the development of diarrhoea in these herds, symptoms seemed not to be caused by insufficient nutrition.

Potential associations between consistency of faeces on day one and the development of NNPDS were of major interest in this study, since a previous study suggested liquid faeces at birth to be a normal finding in these herds [6]. The present study showed that many piglets (46%) having liquid faeces at birth did not develop NNPDS, and that consistency of faeces at birth was only a minor risk factor for developing NNPDS. The decision to of the study to draw a sharp line between day one liquid faeces and day two NNPDS may be problematic, since so many piglets (20-50% of piglets within herds) were found to experience the first symptoms of NNPDS on day 2. Some of these piglets (perhaps especially within Herd 2) probably experienced the first symptoms of NNPDS on day one. The decision to draw this sharp line was made on practical grounds, in order to be able to evaluate the hypothesis of liquid faeces on the day of birth being unrelated to the syndrome. Since the study did (weakly) associate liquid faeces day one with the development of NNPDS, future studies should not rule out that NNPDS sometimes starts on the day of birth.

Naturally, the overall limitation of the study is the lacking definition on NNPDS. Thus, it is important to underline that the conclusions from this study may not apply to all cases of NNPDS, since they were drawn from findings in four herds only. However, the four herds were all thoroughly investigated in terms of possible infectious aetiologies, and none of them were diagnosed with any well-known agent to explain the symptoms. Therefore, it presently seems fair to consider the diarrhoeal outbreaks in these herds to represent NNPDS.

## Conclusions

The prevalence and the duration of diarrhoea as well as the tendency of diarrhoea to cluster within litters differed much between herds diagnosed with NNPDS. In most cases, symptoms started on the second day of life, but in 25-50% of cases within herds symptoms started on the fourth or fifth day of life. The duration of diarrhoea was most often one to two days.

Herd of origin and sow-parity were the most important factors associated with the development of NNPDS, whereas birth weight and faecal consistency on the day of birth were less important risk factors. The study did not point out other sow-level risk factors than parity. The reason for the more severe outbreak of NNPDS in Herd 1 was not explained by factors addressed in this study.

The general hypothesis of liquid faeces day one to be unrelated to the syndrome did not hold true. However, taking all results together, it seems that a liquid consistency faeces at birth is sometimes a harmless phenomenon, unrelated to NNPDS.

The study did not evaluate factors associated with the high prolificacy of Danish genetics (such as longer duration of farrowing) or herd-factors associated with NNPDS. Further studies are needed to look into these aspects.

## Methods

### Ethical approval

The study was conducted in accordance with the guidelines of the Danish Ministry of Justice with respect to animal experimentation and care of animals under study. According to Danish legislation this type of study does not require ethical approval.

### Selection of herds

Four well-managed conventional herds were selected based on these criteria: 1) Persistent problems with diarrhoea during the first week of life with a poor response to antibiotic treatment, 2) Vaccination of sows against *Escherichia coli* and *Clostridium perfringens* type C, 3) Failure of preventive management interventions, 4) PRRS negative farrowing unit as demonstrated in blood samples tested by ELISA/IPT or PCR and 5) Negative results of routine diagnostic examination for enteritis in five piglets. All herds had a history of neonatal diarrhoea for a period of at least one year. Detailed interviews with herd owners, local veterinarians and feed consultants as well as preliminary herd visits were performed in order to exclude herds with obvious management related problems. Prior to the onset of investigations, herd-managers were instructed to carry out prevalence counts of diarrhoea in order to document a relatively high and constant prevalence of diarrhoea (rough estimates – data not shown). Furthermore, herds were instructed not to change any routines before, during or immediately after the study period. After the study periods, follow-up interviews were carried out in order to evaluate if the clinical picture had changed after leaving the herds. Herd 1, 2, 3 and 4 were investigated in January, March, May and July of 2011, respectively.

Diagnostic examinations on a total of 101 Case- and Control piglets from these herds ruled out that well-known infectious agents (enterotoxigenic *E. coli*, *Clostridium perfringens* type C, rotavirus A, coronavirus and *Cystoisospora suis*) could explain the aetiology of diarrhoea. Furthermore, previous studies suggested that neither *Clostridium perfringens* type A, *Clostridium difficile*, *Strongyloides ransomi*, *Giardia spp* nor *Cryptosporidium spp* was involved in the diarrhoeal outbreaks. Gross-pathologically, affected piglets were characterized by flaccidity of intestines with no mucosal lesions and milk-filled stomachs [2].

### Study design and case-definition

The study was carried out as a cross-sectional study with follow-up during the first five days of piglets’ lives in four herds previously diagnosed with NNPDS [2]. On each day of the study, rectal swabs were used to evaluate whether piglets were diarrhoeic (liquid or watery consistency of faeces) or not (creamy, firm and solid consistencies or absence of faeces on swab). NNPDS was defined as diarrhoea at some point during the second to fifth day of life (day one was hypothesized not to be part of the syndrome and piglets were only evaluated until the fifth day of life). Clinical explanatory variables in sows and piglets were registered on the day of parturition/birth (day one).

### Inclusion of sows and piglets

In each herd, approximately 20 sows (6–8 per day) from one farrowing batch were selected on the day of parturition. All selected sows were situated in the same farrowing section. In herds predominantly experiencing problems in first parity litters (Herd 2 and 4), first parity sows were given high priority in the inclusion procedure. Otherwise, the sows that first finished farrowing on the major farrowing days were selected. At selection, litters were standardized to11 (Herd 1 and Herd 3) or 12 piglets (Herd 2 and Herd 4). Piglets to stay in the litters were selected by simple random sampling among littermates having a birth weight above 800 grams. Smaller piglets were excluded, since they were not expected to be able to survive among large litter-mates. All included piglets were kept in their original litters during the whole study period in order to be able to recognize sow-effects. During the study, selected piglets were euthanized for diagnostic purposes. Piglets euthanized without symptoms (Control piglets for the Case–Control study) were removed from data. Data used in the description of duration of symptoms only included piglets still present in the herds on day five of life.

### Treatments during the study period

No preventive antibiotic medication of sows was given. Injection of oxytocin postpartum was accepted if recommended by the local veterinarian. Medical treatment of sows was carried out according to individual herd routines and involved antibiotics and NSAIDs. Decisions to treat were made by the herd-staff and not based on clinical registrations made in the study.

As a general rule, antibiotic treatment of piglets was not accepted within the first three days of life. Later in the study period, clinical diseases were treated according to individual herd routines. Non-antibiotic oral supplements to piglets were allowed during the whole study period.

### Clinical examinations on day one

For each sow, parity, litter size and number of stillborn were registered. All sows were clinically examined between 5 and 20 hours postpartum. The clinical examination included assessment of udder, legs and vulva and registration of rectal temperature. Mastitis was recorded when one or more udder sections were firm, red or sore at palpation. Leg problems were recorded when sows were unwilling to bear equal weight on all legs or evaded palpation of legs or hooves. An excess of unclear or foul-smelling discharge from vulva was recorded as vulva discharge.

Piglets were weighed. The presence of hollow flanks, protruding ribs, rough hair coats, dehydration (lack of skin-elasticity and sunken eye-balls), skin-abrasions on fore-knees and faecal staining of perineum was dichotomously recorded. Faecal staining was assessed within a diameter of one cm around anus. By use of rectal swabs, consistency of faeces was evaluated as either normal (creamy, firm and solid consistencies or absence of faeces on swab) or liquid (liquid or watery consistencies).

All procedures in the herds were carried out by the same person.

### Statistical analyses

Generalized linear mixed models with litter as random effect were used to evaluate potential risk factors for NNPDS. Due to a low prevalence of clinical disease in sows, all disease variables (mastitis, vulva discharge, leg problems and fever) were combined into one. An overview of all risk factors included in the analyses is given in Table 7.

**Table 7:** Description of risk factors evaluated in the study

**Risk-factors**	**Level**	**Categorization**
**Herd**^**1**^	1,2,3,4	
**Sow-related factors**		
Parity	Young	1st parity
	Mature	2nd-7th parity
Litter size	Large^2^	Gilts: >15 piglets, Sows: >18 piglets
	Small	Gilts: <16 piglets, Sows: < 19 piglets
Stillborn	Many	>1 piglet
	Few	0-1 piglets
Clinical disease	Yes	Mastitis and/or temp > 39.5°C and/or leg problems and/or vulva discharge^3^
	No	None of the above
**Piglet-related factors**^**4**^		
Gender	Male	
	Female	
Birth weight	Continuous scale	
Faecal consistency	Liquid	Watery or liquid consistency of rectal contents
	Normal	Creamy, firm or solid consistency of rectal contents and if no faeces on swab
Flanks	Hollow	Area behind ribs turned inwards
	Normal	Area behind ribs followed the line of the ribs
Hair coat	Rough	Hair coat appeared dull
	Normal	Hair coat did not appear dull

^1^No herd effect was included in the overall parity-separated models.

^2^Litters above mean size of the parity group in question were considered large.

^3^Mastitis: One or more udder section hard, red or sore when palpated. Leg problems: The sow was unwilling to bear weight on all legs or sore at palpation. Vulva discharge: An excess of unclear or foul-smelling discharge.

^4^Protruding ribs, fore-knee abrasions and dehydration were too low prevalent to be included in the statistical models.

All clinical signs were assessed on the day of parturition/birth.

Models were fit in R [12] using the lme4 package [13]. Model reduction was carried out using stepwise backwards elimination, removing variables with p > 0.05. Confounding was assessed by taking out and re-entering variables into the final models one by one and check for biologically important changes of estimates. Interaction terms were not included due to problems with complete separation, caused by sparse data. The linearity of birth weight at the log odds scale for NNPDS was assessed by transforming birth weight into a categorical variable based on quartiles and then verify that a decreasing trend of the estimates for the levels of the categorical variable was observed.

Population average Odds Ratios (OR_PA_) were calculated using the following formula:

$${\mathrm{OR}}_{\mathrm{PA}}=\exp\left(\beta_{\mathrm{SS}}\right)/\mathrm{sqrt}\left(1+0.346*{\delta^{2}}_{\mathrm{litter}}\right)$$

where β_SS_ is the Subject Specific regression coefficient and δ^2^_litter_ is the litter variance. The constant 0.346 is an approximation of the residual variance [14].

Population averaged, rather than cluster specific estimates of OR’s were used, since the study aimed at drawing general rather than litter-specific conclusions.

Pairwise post-hoc comparisons within significant variables with more than two levels were carried out using the lsmeans package in R [15].

Since data was limited, modelling was performed using a three-step procedure in order to be able to identify all associations of practical interest.

Step 1: Two separate models – one for first parity piglets and one for piglets born by mature (2nd-7th parity) sows -were run to identify overall sow-related risk-factors for NNPDS. The separation into two models was done in order to be able to recognize possible differences in sow-effects in first parity vs. mature sows. Furthermore, since first parity sows had smaller litters, different cut-offs were needed in the evaluation of the effect of litter size. In each model, litter size was inserted dichotomously with cut-off at the mean litter size in question (15 and 18, respectively). Herds were not included in these models, in order to avoid overlooking any subtle sow-effects.

Step 2: If the results of the parity-specific models in step 2 were alike, an overall model including parity and herd-effects was generated.

Step 3: Separate models for each herd were used to evaluate risk factors and random litter effects within the separate herds. These models were furthermore used to properly evaluate piglet-level risk-factors (due to the previously mentioned inability to include interaction terms in Step 2).

## Competing interests

The authors declare that they have no competing interests.

## Authors’ contributions

All authors contributed to the design of the study. JPN contributed to the practical design of the field studies and NT contributed to decisions on the statistical approach of the study. Inclusion of herds, clinical examination in the herds and statistical analyses were performed by HK. All authors participated in drafting the manuscript and proofreading of the manuscript. All authors read and approved the final manuscript.

## References

[B1] Kongsted H Spædgrisediarré i Danmark Anno 2013 (Danish) http://vsp.lf.dk/~/media/Files/PDF%20-%20Publikationer/Erfaringer%202013/Erfaring_1320_Sp%C3%A6dgrisediarre_ i_Danmark_anno_2013.ashx

[B2] Kongsted H Jonach B Haugegaard S Angen O Jorsal SE Kokotovic B Larsen LE Jensen TK Nielsen JP Microbiological, pathological and histological findings in four Danish pig herds affected by a new neonatal diarrhoea syndrome BMC Vet Res 2013 9 206 10.1186/1746-6148-9-206 24119974 PMC3852778

[B3] Svensmark B Jorsal SE Nielsen K Willeberg P Epidemiological studies of piglet diarrhoea in intensively managed Danish sow herds. I. Pre-weaning diarrhoea Acta Vet Scand 1989 30 1 43 53 2782232 10.1186/BF03548067 PMC8142215

[B4] Wittum TE Dewey CE Hurd HS Dargatz DA Hill GW Herd- and litter-level factors associated with the incidence of diarrhea morbidity and mortality in piglets 1 to 3 days of age Swine Health Prod 1995 3 3 99 104

[B5] Lingaas F Epidemiological and genetical studies in Norwegian pig herds. IV. Breed effects, recurrence of disease, and relationship between disease and some performance traits Acta Vet Scand 1991 32 1 107 114 1950843 10.1186/BF03547002 PMC8127935

[B6] Kongsted H Stege H Toft N Nielsen JP The effect of New Neonatal porcine Diarrhoea Syndrome on average daily gain and mortality in four Danish pig herds BMC Vet Res 2014 10 90 10.1186/1746-6148-10-90 24755093 PMC3999350

[B7] Lingaas F Ronningen K Epidemiological and genetical studies in Norwegian pig herds. II. Overall disease incidence and seasonal variation Acta Vet Scand 1991 32 1 89 96 1950855 10.1186/BF03547000 PMC8127902

[B8] Sialelli J Lautrou Y Oswald I Quiniou N Peut-on établir une relation entre les charactéristiques de la trui et de sa portée et l’apparition des diarrhées néonathales? [abstract] Journées Recherche Porcine 2009 41 167 172

[B9] Fairbrother JM Gyles CL Zimmerman JJ, Karriker LA, Ramirez A, Schwartz KJ, Stevenson GW Colibacillosis Diseases of Swine 2012 10 Iowa, USA: Wiley-Blackwell 723 749

[B10] Beyer M Jentsch W Kuhla S Wittenburg H Kreienbring F Scholze H Rudolph PE Metges CC Effects of dietary energy intake during gestation and lactation on milk yield and composition of first, second and fourth parity sows Arch Anim Nutr 2007 61 6 452 466 10.1080/17450390701563433 18069617

[B11] Pedersen LJ Jensen T Effects of late introduction of sows to two farrowing environments on the progress of farrowing and maternal behavior J Anim Sci 2008 86 10 2730 2737 10.2527/jas.2007-0749 18469055

[B12] CoreTeam R R: A Language and Environment for Statistical Computing 2013 Vienna, Austria: R foundation for statistical computing http://www.Rproject.org/

[B13] Bates D Maechler M Bolker Blme4: Linear Mixed-Effects Models Using S4classes 2013 http://www.CRAN.R-project.org/package=lme4

[B14] Dohoo I Martin W Stryhn H Veterinary Epidemiologic Research: 2nd ed 2009 Charlottetown, Prince Edward Island, Canada: VER Inc

[B15] Lenth RVlsmeans: Least-Squares Means 2013 http://www.CRAN.R-project.org/package=lsmeans

